# Editorial: Understanding PFO-associated stroke

**DOI:** 10.3389/fneur.2023.1274123

**Published:** 2023-08-24

**Authors:** Theodoros Karapanayiotides, Vasileios-Arsenios Lioutas, Marta Rubiera, Gilles Montalescot, Panayiotis Mitsias

**Affiliations:** ^1^2nd Department of Neurology, Aristotle University of Thessaloniki, School of Medicine, Thessaloniki, Greece; ^2^Division of Vascular Neurology, Department of Neurology, Beth Israel Deaconess Medical Center and Harvard Medical School, Boston, MA, United States; ^3^Stroke Unit, Department of Neurology, Hospital Vall d'Hebron, Universitat Autónoma de Barcelona, Badalona, Spain; ^4^Institut de Cardiologie, Hôpitaux Universitaires Pitié Salpêtrière, Paris, France; ^5^Department of Neurology, School of Medicine, University of Crete, Heraklion, Greece

**Keywords:** stroke, cryptogenic stroke, PFO (patent foramen ovale), right to left cardiac shunt, paradoxical embolism

It has been well-established for more than two decades that the prevalence of patent foramen ovale (PFO) in patients with cryptogenic stroke is considerably higher than that in the general population (1). However, the prevalence of PFO and the right-to-left size of the shunt in the general population and in stroke patients may fluctuate widely depending on the diagnostic modality, the protocols used, age, and little investigated racial or ethnic discrepancies (2). The term PFO-associated stroke (PFOaS) has been recently introduced as a distinct causative mechanism of ischemic stroke and a causal likelihood classification system has been proposed to optimize patient selection for PFO closure (3, 4). PFOaSs are usually associated with paradoxical embolism or with *in situ* formation of thrombi in the PFO tunnel (5). However, in most cases, PFOaS is a diagnosis of speculation. PFO is diligently investigated for in cryptogenic stroke; however, PFOs of diverse sizes may be found in more than one-third of normal individuals (2). Therefore, to assign stroke causality one needs to take into account several co-existing -yet little-investigated factors- such as anatomic and functional PFO characteristics, PFOaS mechanisms apart from paradoxical embolism, prothrombotic conditions, asymptomatic pulmonary embolism, age, and vascular risk factors. All these features may have a significant impact on the individualization of PFOaS risk assessment and management. In this Research Topic, we collected a number of well-conducted original studies and state-of-the-art narrative reviews in an attempt to elucidate controversial aspects of PFOaS.

In an elegant review, Huber et al. highlight all the current challenges in the management of patients with PFOaS. They underscore the lack of solid data supporting the prevailing pathophysiological concept of paradoxical embolism associated with deep venous thrombosis. The latter, being notoriously difficult to document, was not taken into account by any of the trials which documented the superiority of PFO closure over the best medical treatment. The alternative assumption of *in situ* PFO thrombogenicity is equally difficult to prove. The optimal secondary prevention of patients with TIAs who harbor a PFO remains unknown since these patients have been excluded from most randomized closure trials. Furthermore, the concept of PFOaS in older patients is even more obscure and the optimal secondary prevention strategies need to be addressed by future trials.

The complexity of PFO pathophysiology and the association of PFO with diseases beyond stroke such as migraine is eloquently reviewed by Shi et al. PFO may be associated with abnormal metabolism of serotonin caused by increased production via activated platelets and decreased lung clearance owing to the right-to-left shunt (RLS). A platelet-derived systemic prothrombotic state coupled with an altered oxidative stress status may lead to PFO-associated micro- or macroembolisms in the brain. Consequently, one would expect the activity of biomarkers associated with antithrombotic properties such as ADAMTS-13 to be lower in PFO carriers prone to DVT and paradoxical embolism. Surprisingly, Grosse et al. suggest that ADAMTS-13 is associated with the presence of vascular risk factors and not with the risk of paradoxical embolism in a small cohort of young patients with PFOaS, underscoring that the complicated interactions of PFO with the mechanisms of thrombosis still remain elusive.

Regarding PFO diagnosis, the importance of ultrasound modalities is paramount. A meta-analysis comparing transthoracic (TTE) with transesophageal echocardiography (TEE) documented the low sensitivity (45%) but very high specificity (>99%) of TTE for the detection of PFO (6). The authors of the European position paper on the management of patients with PFO conducted a meta-analysis of 2,751 patients and established the high accuracy of transcranial Doppler (TCD) compared with TEE (sensitivity: 94%, specificity: 92%, area under the receiver operating curve: 0.97) (7). Considering that TEE is invasive and not readily available across different health systems, Yao et al. evaluate synchronous testing with TTE and TCD in a prospective study. They conclude that their combined approach improves the total positive and detection rate of moderate-to-large RLS compared with the use of the individual tests. Compared with TEE, synchronous multimode ultrasound had a sensitivity of 87.5% and a specificity of 60.6% for PFO diagnosis. In the same direction of restrained use of TEE resources in patients with cryptogenic stroke < 60 years old, Mayerhofer et al. document that combined non-invasive ultrasound reaches a sensitivity of 98.0% and a negative predictive value of 97.1% for therapy-relevant findings in TEE (PFO or aortic arch atheromatosis). Thus, in younger patients with cryptogenic stroke, if both TCD and ultrasound of the neck vessels are without significant findings, TEE may be skipped without significant risk. TEE may be considered the “gold standard” for PFO detection but there are good arguments to suggest that TEE is a standard of uncertain validity (2). Major limitations of TEE include that patients are prone not to be able to perform an effective Valsalva maneuver due to sedation-induced poor cooperation and to the presence of the TEE probe in the esophagus. Zhu et al. show that calf muscle pump tensing is a novel and effective provocative maneuver that may increase the diagnostic yield in patients undergoing TEE for PFO diagnosis.

PFOaS recurrence has been little studied and risk factors for recurrence are mainly considered to pertain to the anatomic characteristics of PFO. Wu et al. follow a cohort of PFOaS patients for 5 years and construct a practical nomogram predicting stroke recurrence based exclusively on three serum biomarkers (homocysteine, hsCRP, and albumin). The nomogram is subsequently tested in a validation cohort and shows good discrimination (2-year AUC, 0.839; 5-year AUC, 0.990). The study highlights the importance of systemic factors associated with endothelial dysfunction, inflammation, and oxidative stress in interacting with PFO and leading to stroke recurrence.

Finally, Laghlam et al. provide a view of PFOaS from a cardiosurgical perspective in a large retrospective study. They identify risk factors associated with PFO reopening during cardiac surgery and they evaluate the relation between PFO reopening and post-operative complications and stroke. They show that PFO reopening during cardiac surgery occurs in only 2.1% of patients but postoperative PFO reopening is a strong predictor of postoperative stroke (adjusted OR: 3.5).

In conclusion, more th an 150 years after Julius Cohnheim described the first case of fatal paradoxical embolism through a PFO to the middle cerebral artery, PFOaS remains, in the majority of cases, a presumptive diagnosis with many fields open to research. Despite significant advances in secondary prevention, the major factors that render a pathology present in more than one-third of the general population a source of brain ischemia are still to be determined.

## Author contributions

TK: Writing—original draft. V-AL: Writing—review and editing. MR: Writing—review and editing. GM: Writing—review and editing. PM: Writing—review and editing.
